# The prognostic value of point-of-care blood glucose and lactate in canine and feline pyothorax: a retrospective analysis

**DOI:** 10.3389/fvets.2025.1581701

**Published:** 2025-05-09

**Authors:** Lei Zhang, Megan Grobman

**Affiliations:** ^1^Department of Veterinary Clinical Medicine, University of Illinois Urbana-Champaign College of Veterinary Medicine, Urbana, IL, United States; ^2^Department of Clinical Sciences, Auburn University College of Veterinary Medicine, Auburn, AL, United States

**Keywords:** pyothorax, hyperlactatemia, lactate, glucose, prognostic, point-of-care

## Abstract

**Introduction:**

Blood lactate and glucose are recognized as prognostic markers in various diseases in human and animals, yet studies evaluating their prognostic utility in canine and feline pyothorax are limited. This study aimed to investigate the prognostic utility of point-of-care (POC) lactate and glucose measurements, and identify clinical factors associated with survival in dogs and cats with pyothorax.

**Methods:**

A database search identified canine and feline pyothorax cases presenting to Auburn University between 2013 and 2023. Forty-three dogs and eight cats diagnosed with pyothorax were retrospectively enrolled. Baseline characteristics, clinicopathological data, and diagnostic findings were obtained from the medical records. POC blood glucose and lactate, recorded on admission and the following morning were documented, and lactate delta and clearance were calculated. Data on treatment was also collected. Additional outcome measures included survival and duration of hospitalization.

**Results:**

In dogs, non-survivors had significantly higher POC lactate concentrations on admission compared to survivors (3.4 mmol/L vs. 2.0 mmol/L, *p* = 0.023). Band neutrophil count was associated with in-hospital mortality at a univariable level (*p* = 0.03, OR 1.4). Stepwise Cox regression showed total solids and lactate concentration on admission as independent predictors of outcome. The area under the curve (AUC; 95% CI) for predicting in-hospital mortality based on lactate was 0.724 (0.568–0.879).

**Conclusion:**

In dogs with pyothorax, admission POC blood lactate concentration may serve as valuable prognostic tool to guide clinical management decisions, and an increased band neutrophil count is associated with poorer outcomes. Further large-scale studies are needed to confirm these findings.

## 1 Introduction

Pyothorax is characterized by the presence of purulent exudate in the pleural space (1). Despite the numerous potential routes of infection reported in previous studies, the underlying cause is usually not determined (1, 2). Medical management is the primary treatment for pyothorax (3, 4), though surgical intervention may be warranted in cases involving structural diseases or when medical management fails (4–6). Despite these interventions, prognosis is variable (7, 8), with the highest mortality rate occurring within the first 48 h of hospitalization (9, 10). Point-of-care biomarkers may help serve as readily available, early indicators of diseases severity and prognosis during the acute hospitalization period in dogs and cats with pyothorax.

Plasma lactate and glucose concentrations are valuable triage parameters and established prognostic biomarkers of mortality in critically ill patients. In human medicine, increased lactate concentrations at admission, persistently high lactate concentrations, or low lactate clearance are associated with severe disease and increased hospital mortality (11–13). In veterinary medicine, multiple studies have demonstrated the prognostic value of lactate concentration in dogs and cats (14–17). In dogs presented to the emergency room with respiratory disease, hyperlactatemia was significantly associated with higher mortality (14). Abnormal glucose concentrations were associated with severity of illness and increased mortality in hospitalized human patients (18). Similar findings have been identified in small animal patients where dysglycemia, (e.g., hyperglycemia or hypoglycemia) was found to be linked to disease severity and a higher mortality rate in dogs and cats (19–22).

Dysglycemia and hyperlactatemia are not uncommon in dogs and cats with pyothorax (3, 7, 9). Despite the clinical significance of this condition, point-of-care biomarkers such as glucose, lactate remain unstudied. The primary objective of this retrospective study was to assess the association between point-of-care blood lactate, lactate clearance, and blood glucose concentrations upon admission and during the first 24 h of hospitalization, and disease outcomes in a population of dogs and cats with pyothorax. The secondary objectives included reporting signalment, history, clinical signs and physical examination findings, diagnostic results, and in-hospital treatment protocols, and investigating their relationship with survival. We hypothesized dysglycemia, hyperlactatemia, and or low lactate clearance would be associated with significantly higher mortality in dogs and cats with pyothorax.

## 2 Methods

### 2.1 Case selection

The medical record database of the Auburn University Veterinary Teaching Hospital (AUVTH) was searched with a keyword diagnosis of pyothorax in all dogs and cats admitted to the emergency, internal medicine, soft tissue surgery, and oncology services over a 10-year period (2013 to 2023).

All medical records were reviewed, and the diagnosis of pyothorax was confirmed based on at least one of the following criteria:

1. Pleural fluid cytology consistent with a suppurative exudate with protein concentrations ≥2.5 g/dL or cell count ≥5,000/uL (2).

a. Effusions were described as septic when bacteria were identified on cytology by a veterinary clinical pathologist.

2. Positive aerobic and/or anerobic culture from pleural fluid.
3. Tissue analysis (histopathology, cytology, or culture) from surgical samples showing suppurative inflammation and or positive bacterial culture.
4. Necropsy findings consistent with pyothorax.

To be eligible for inclusion, dogs and cats were required to have had an admission blood lactate and glucose concentrations that were measured with a point-of-care analyzer. Cats and dogs with neoplastic or aseptic chylous effusions or cats with feline infectious peritonitis (FIP) effusions diagnosed on cytology were excluded.

### 2.2 Data collection

Variables that were collected from the medical records included the patients’ signalment (e.g., age, sex, reproductive status, breed), presenting complaints, duration of clinical signs prior to presentation, body weight, and vital parameters (e.g., respiratory rate, heart rate, temperature, and blood pressure). Also recorded were physical exam findings (e.g., respiratory effort/pattern, hydration status) and Quick Assessment Tests (QATs) including POC blood glucose, POC blood lactate, packed cell volume (PCV), and total solids (TS).

The medical records were also reviewed for the Thoracic Point of Care Ultrasound (T-POCUS) findings, characterization of pleural effusion (involved hemithorax [right, left, both], volume), diagnostic imaging findings (e.g., radiographs, ultrasound, and computed tomography [CT]), hematocrit (HCT), white blood cells (WBCs), band neutrophil count, platelet count, and body cavity fluid analysis (e.g., microscopic exam findings, cytology, culture). Other diagnostic tests such as tissue biopsy (histopathology, cytology, culture), and necropsy results were assessed where available. Treatment was categorized as either medical or surgical management. Further information regarding administered therapy including thoracostomy tube placement and its indwelling duration, and in-hospital antibiotic administration were collected. Recheck blood lactate and glucose concentrations obtained at 8 AM the following morning were also recorded where available.

The underlying cause of pyothorax was recorded when identified. Patient outcome was reported as either survivor or non-survivor. Non-survivors were further described as euthanasia and cardiopulmonary arrest. The length of hospitalization was documented if patients were alive at discharge; the duration of hospitalization before death was recorded for non-survivors.

### 2.3 Statistical analysis

For data analysis, tachypnea was defined as a resting respiratory rate > 40 breaths per minute (brpm). Hypothermia and hyperthermia were defined as a temperature < 100.5°F (38.1°C) or > 102.5°F (39.2°C), respectively. Tachycardia was defined as a heart rate (HR) > 180 beats/min (bpm) in dogs and > 220 bpm in cats, and bradycardia as a HR < 60 bpm in dogs and < 160 bpm in cats. Hypertension and hypotension were defined as systolic blood pressure < 90 mmHg and > 140 mmHg, respectively. Hyperlactatemia was defined as blood lactate > 2.5 mmol/L. Hyperglycemia was defined as blood glucose > 180 mg/dL, and hypoglycemia as blood glucose < 60 mg/dL (23). Size categories were defined based on body weight, < 10 kg as small, > 10 kg and < 25 kg as medium, > 25 kg as large size (24). Lactate delta and lactate clearance were calculated when available using the following formulas (25):


LactateDelta=Lactate2−Lactate1

$$\mathrm{Lactate}\;\mathrm{Clearance}=\frac{\left(\mathrm{Lactate}\;1-\mathrm{Lactate}\;2\right)}{\mathrm{Lactate}\;1}\times 100$$

Lactate 1 is the POC blood lactate concentration on admission; Lactate 2 is the POC lactate concentration at 8 AM the following morning. A negative lactate clearance indicates increased lactate over time, while a positive lactate clearance indicates a decreased lactate over time.

Variables with more than 10% missing data were excluded from statistical analysis to minimize potential bias; however, descriptive data were provided for these variables. Due to the small number of cats in this study, which limits the power to detect significant differences and associations, statistical analysis was not performed. Instead, descriptive data were used to provide an overview of clinical characteristics, diagnostic findings, and outcomes in cats.

Data were assessed for normality by Shapiro–Wilk test. Mean ± standard deviations (SD) were used to describe parametric data, while median with first and third quartiles were used to describe nonparametric data for dogs and cats. Parametric data were analyzed by two-tailed Student t test. Nonparametric data were analyzed utilizing Mann–Whitney U test. Categorical variables were reported as the numbers and percentages and compared by the chi-squared analysis or Fisher’s exact test. Statistical significance was set at *p* < 0.05. The Benjamini-Hochberg Procedure was used to adjust *p* values when comparing variables of diagnostic findings (clinicopathological data, thoracic fluid analysis, diagnostic imaging findings, and treatments), with a false detect rate (FDR) threshold set at 0.05.

The univariable logistic regression analysis was used to investigate the associations of variables with outcome. The Stepwise Cox regression analysis was used to identify predictors of mortality. Receiver-operating curves (ROCs) with the corresponding area under curve (AUC) were applied to evaluate the predictive ability of selected variables. Kaplan–Meier survival curves were applied to evaluate survival in dogs with increased and normal lactate concentrations. Data were analyzed with commercially available software (Microsoft Excel Version 16.92, IBM SPSS Statistics 29.0.0.0, and RStudio Version 2023.12.0).

## 3 Results

A medical record search yielded 47 dogs and 11 cats with a diagnosis of pyothorax. QATs had not been performed in 4 dogs and 3 cats. Consequently, 43 dogs and 8 cats were included in the study.

### 3.1 Clinical characteristics

#### 3.1.1 Dogs

Dogs’ baseline characteristics and pertinent historical data are summarized in Table 1. In dogs, non-survivors (*n* = 12, 70 ± 19.8 brpm) had a higher mean respiratory rate compared to survivors (*n* = 16, 53.4 ± 18.0 brpm); however, respiratory rate was not specified for over 30% of the dogs. As such, its significance was not evaluated. No significant differences were found between the survivor and non-survivor groups in terms of signalment, age, size, sex, or reproductive status. No clinical signs were significantly associated with death, and the duration of clinical signs prior to presentation did not differ significantly between survivor and non-survivor groups. No other vital parameters or physical examination findings were significantly different between groups.

**Table 1:** Dogs’ characteristics and pertinent historical summary.

Variables	Overall (*n* = 43)	Survivor (*n* = 27)	Non-survivor (*n* = 16)	*p*-value
Age (month)	42.2 (21.6, 97.4)	38.6 (21.6, 68.3)	106.2 (20.9, 109.6)	0.119
Male/Female	20/23 (47%/53%)	13/14 (48%/52%)	7/9 (44%/56%)	0.780
Intact/Castrated	19/24 (44%/46%)	13/14 (48%/52%)	6/10 (38%/63%)	0.497
Size				
Small	5 (12%)	2 (7%)	3 (19%)	0.466
Medium	14 (33%)	10 (37%)	4 (25%)	
Large	24 (56%)	15 (56%)	9 (56%)	
Breed				
Mixed breed	6 (14%)	5 (19%)	1 (6%)	-
German Shepherd Dog	4 (9%)	2 (7%)	2 (13%)	
German Shorthaired Pointer	3 (7%)	3 (11%)	0	
Labrador Retrievers	3 (7%)	1 (4%)	2 (13%)	
English Pointers	2 (5%)	1 (4%)	1 (6%)	
American Bulldog	2 (5%)	1 (4%)	1 (6%)	
Old English Sheepdog	2 (5%)	1 (4%)	1 (6%)	
Others (*n* = 1)	21 (49%)	13 (48%)	8 (50%)	
Presenting complaints				
Respiratory abnormalities	27 (63%)	15 (56%)	12 (75%)	0.202
Anorexia/hyporexia	26 (60%)	19 (70%)	7 (44%)	0.084
Lethargy/weakness/exercise intolerance	19 (44%)	13 (48%)	6 (38%)	0.497
Coughing	12 (28%)	10 (37%)	2 (13%)	0.167
GI signs	9 (21%)	5 (19%)	4 (25%)	0.907
Pleural effusion	8 (19%)	5 (19%)	3 (19%)	0.699
Fever	5 (12%)	5 (19%)	-	0.181
Weight loss	4 (9%)	3 (11%)	1 (6%)	0.990
Duration of CS (days) (*n* = 42)	4 (1–49)	5 (1–49)	4 (2–14) (*n* = 15)	0.327
Weight (kg)	26.34 ± 2.06	27.27 ± 12.45	24.77 ± 15.41	0.563
T (**°**F)	102.5 ± 2.1	102.4 ± 2.1	102.5 ± 2.1	0.877
T > 102.5°F	18 (42%)	12 (44%)	6 (38%)	0.840
100.5 ≤ T ≤ 102.5°F	19 (44%)	11 (41%)	8 (50%)	
T < 100.5°F	6 (14%)	4 (15%)	2 (13%)	
PR (bpm)	139.4 ± 27.5	136.6 ± 23.8	144.2 ± 33.2	0.430
PR ≤ 180 bpm	40 (93%)	26 (96.3%)	14 (87.5%)	0.274
PR > 180 bpm	3 (7%)	1 (3.7%)	2 (12.5%)	
RR (brpm) (*n* = 28)	60.5 ± 20.2	53.4 ± 18.0 (*n* = 16)	70 ± 19.8 (*n* = 12)	-
Pant	15 (35%)	11 (41%)	4 (25%)	0.283
RR > 40 brpm	23 (53%)	12 (44%)	11 (69%)	
RR ≤ 40 brpm	5 (12%)	4 (15%)	1 (6%)	
SBP (mmHg) (*n* = 31)	135.6 ± 6.7	144.1 ± 23.3 (*n* = 19)	122.2 ± 51.0 (*n* = 12)	-
Hypertension	14 (45%)	10 (53%)	4 (33%)	-
Nomotension	16 (52%)	9 (47%)	7 (58%)	
Hypotension	1 (3%)	-	1 (8%)	
Hydration status (*n* = 12)				
Euhydrated	1 (8%)	1 (14%)	-	-
<5%	2 (17%)	1 (14%)	1 (20%)	
5 to 7%	7 (58%)	4 (57%)	3 (60%)	
>7%	2 (17%)	1 (14%)	1 (20%)	
Respiratory effort (*n* = 39)				
Normal	8 (21%)	7 (29%)	1 (14%)	0.090
Increased	31 (79%)	17 (71%)	7 (93%)	

Parametric data are presented as mean ± SD; Non-parametric data are presented as median with first and third quartiles (P25, P75); Categorical data was presented as numbers and percentages.

#### 3.1.2 Cats

Cats that survived to discharge had a higher average temperature (mean 102.37 ± 1.47°F) compared to non-survivors (mean 99.17 ± 1.10°F). All hypothermic cats did not survive. An overview of clinical characteristics, diagnostic findings, and outcomes in cats are provided in Table 2.

**Table 2:** Comparison of variables in cats with pyothorax between survivors and non-survivors.

Variables	Overall (*n* = 8)	Survivor (*n* = 4)	Non-survivor (*n* = 4)
Breed			
DSH	6	4 (100%)	2 (50%)
Maine Coon	1	0	1 (25%)
Ragdoll	1	0	1 (25%)
Male/Female	5/3 (62.5%/37.5%)	3/1 (75%/25%)	2/2 (50%/50%)
Intact/Castrated	3/5 (37.5%/62.5%)	1/3 (25%/75%)	2/2 (50%/50%)
Age (mo) (*n* = 7)	25 (8, 183)	19 (8, 61)	38 (15, 183) (*n* = 3)
Presenting complaints			
Anorexia/hyporexia	5 (63%)	3 (75%)	2 (50%)
Respiratory abnormalities	7 (88%)	4 (100%)	3 (75%)
Lethargy	4 (50%)	2 (50%)	2 (50%)
Fever	1 (13%)	0	1 (25%)
Pleural effusion	2 (25%)	0	2 (50%)
GI signs	1 (13%)	1 (25%)	0
Weight loss	1 (13%)	0	1 (25%)
Duration of CS (days) (*n* = 6)	1.5 (1, 14)	1 (1, 14) (*n* = 3)	2 (1, 4) (*n* = 3)
Weight (kg)	4.65 (3.5, 8.4)	3.95 (3.5, 5.5)	5.1 (4.1, 8.4)
T (°F) (*n* = 7)	100.54 ± 2.06	102.37 ± 1.47 (*n* = 3)	99.17 ± 1.10
PR (bpm)	181.5 ± 28.02	185.00 ± 41.23	178.00 ± 9.93
RR (brpm) (*n* = 28)	57.75 ± 10.05	55.50 ± 12.48	60.00 ± 8.16
Clinicopathologic variables			
HCT (%, *n* = 5) [38.7–59.2%]	38.9 (24.3, 49)	37.25 (24.3, 49)	38.9 (*n* = 1)
Decreased HCT	2 (40%)	2 (50%)	0
WBCs (x10^3/uL, *n* = 5) [5.09–17.41]	27.97 (17.57, 88.87)	26.97 (17.57, 88.87)	27.97 (*n* = 1)
Increased WBCs	5 (100%)	4 (100%)	1 (100%)
Band neutrophils (x10^3/uL, *n* = 5) [0–0.3]	8.73 (0.699, 17.77)	8.05 (0.699, 17.77)	9.789 (*n* = 1)
Increased band neutrophils	5 (100%)	4 (100%)	1 (100%)
Platelet count (x10^3/uL, *n* = 5) [52–518]	379 (170, 415)	329.5 (170, 415)	379 (*n* = 1)
Body cavity fluid analysis (*n* = 7)			
Possible bacteria cytologically	1 (14%)	1 (25%)	0
Positive bacteria cytologically	6 (86%)	3 (75%)	3 (100%) (*n* = 3)
Fluid culture (aerobic) (*n* = 5)			
Positive	3 (60%)	2 (50%) (*n* = 4)	1 (100%) (*n* = 1)
Negative	2 (60%)	2 (50%) (*n* = 4)	0
Fluid culture (anaerobic) (*n* = 4)			
Positive	4 (100%)	3 (100%) (*n* = 3)	1 (100%) (*n* = 1)
Negative	0	0	0
Pleural effusion (side)			
Bilateral	4 (50%)	3 (75%)	1 (25%)
Unilateral	1 (12.5%)	0	1 (25%)
Undefined side	3 (37.5%)	1 (25%)	2 (50%)
Pleural effusion (volume)			
Large	2 (25%)	2 (50%)	0
Mild	1 (12.5%)	0	1 (25%)
Moderate	1 (12.5%)	0	1 (25%)
Undefined	4 (50%)	2 (50%)	2 (50%)
Diagnosis (*n* = 7)			
Pleuritis	4 (57%)	2 (50%)	2 (67%) (*n* = 3)
Pleuropneumonia	2 (29%)	1 (25%)	1 (33%) (*n* = 3)
Pulmonary abscess	1 (14%)	1 (25%)	0
QATs (Quick Assessment Tests)			
PCV 1 (%) (*n* = 7)	46.57 ± 12.79	51.33 ± 11.55 (*n* = 3)	43.00 ± 14.09
TS 1 (g/dl) (*n* = 7)	7 ± 0.5	7.33 ± 0.42 (*n* = 3)	6.75 ± 0.44
BG 1 (mg/dl)	137.75 ± 59.82	158.75 ± 59.58	116.75 ± 60.19
Hyperglycemia 1	1 (12.5%)	1 (25%)	0
Hypoglycemia 1	1 (12.5%)	0	1 (25%)
Normoglycemia 1	6 (75%)	3 (75%)	3 (75%)
Lac 1 (mmol/L)	4.64 ± 4.9	7.02 ± 6.27	2.25 ± 1.24
Hyperlactatemia 1	5 (62.5%)	2 (50%)	3 (75%)
Normolactatemia 1	3 (37.5%)	2 (50%)	1 (25%)
PCV 2 (%) (*n* = 5)	36.6 ± 9.58	38.50 ± 10.61 (*n* = 3)	35.33 ± 11.02 (*n* = 2)
TS 2 (g/dl) (*n* = 5)	5.92 ± 0.82	5.73 ± 0.46 (*n* = 3)	6.20 ± 1.41 (*n* = 2)
BG 2 (mg/dl) (*n* = 5)	134.8 ± 40.23	138.00 ± 54.25 (*n* = 3)	130.00 ± 22.63 (*n* = 2)
Hyperglycemia 2	1 (20%)	1 (33%)	0
Normoglycemia 2	4 (80%)	2 (67%)	2 (100%)
Lac 2 (mmol/L) (*n* = 5)	1.12 ± 0.16	1.13 ± 0.21 (*n* = 3)	1.10 ± 0.14 (*n* = 2)
Lactate delta (mmol/L) (*n* = 2)	−2.2	−1.8 (*n* = 1)	−2.5 (*n* = 1)
Lactate clearance (%) (*n* = 2)	69%	64% (*n* = 1)	74% (*n* = 1)
In-hospital Antibiotics (*n* = 6)			
Ampicillin and sulbactam	5 (83%)	3 (75%)	2 (100%) (*n* = 2)
Enrofloxacin	2 (33%)	1 (25%)	1 (50%) (*n* = 2)
Clindamycin	1 (17%)	1 (25%)	0
Metronidazole	2 (33%)	1 (25%)	1 (50%) (*n* = 2)
Chest tube placement (*n* = 6)			
Bilateral	1 (17%)	1 (25%)	0
Unilateral	5 (83%)	3 (75%)	2 (100%) (*n* = 2)
Outcome			
Length of Hospitalization (days)		6.5 (5, 8) (*n* = 4)	
Duration of Hospitalization (in-hospital death) (days)			1.5 (1, 5) (*n* = 4)

Parametric data are presented as mean ± SD; Non-parametric data are presented as median with minimum and maximum; Categorical data was presented as numbers and percentages.

### 3.2 Diagnostics

#### 3.2.1 Dogs

Hematology results were available for 40 dogs. Nine (22.5%) dogs were considered anemic (survivors [*n* = 5] and non-survivors [*n* = 4]), and 1 (3%) dog had an increased hematocrit (HCT). HCT was significantly (*p* = 0.049) (Table 3) lower in the non-survivor group (mean 39.8% ± 9.6%), compared to the survivor group (mean 48.0% ± 9.8%). A leukocytosis was observed in 16 (40%) dogs and leukopenia in 7 (18%) dogs. Non-survivors had significantly (*p* = 0.022) lower white blood cell counts (median 7.9 ×10^3/uL, 3.9 to 14.0 ×10^3/uL), compared to survivors (median 18.5 ×10^3/uL, 13.1 to 22.2 ×10^3/uL). Band neutrophil counts were increased in 26 (65%) dogs. Non-survivors (median 1.2 ×10^3/uL, 0.5 to 7.4 ×10^3/uL) had significantly (*p* = 0.042) higher band neutrophil counts, compared to survivors (median 0.4 ×10^3/uL, 0 to 1.2 ×10^3/uL). Abnormal platelet counts were recorded in 6 (15%) dogs: thrombocytopenia (*n* = 1) and thrombocytosis (*n* = 5). No significant (*p* = 0.206) difference was found in platelet count between dogs in the survivor versus non-survivor groups.

**Table 3:** Dogs’ clinical data (clinicopathological data, body cavity fluid analysis, CT findings, treatment) summary.

Variables	Overall (*n* = 43)	Survivor (*n* = 27)	Non-survivor (*n* = 16)	*p*-value	Adjusted *p*-value
HCT (%, *n* = 40) [38.7–59.2%]	45.11 ± 10.4	48.0 ± 9.8 (*n* = 26)	39.8 ± 9.6 (*n* = 14)	0.016*	0.049*
Decreased HCT	9 (22.5%)	5 (19%)	4 (29%)	0.629	0.629
Increased HCT	1 (2.5%)	1 (3.8%)	-		
WBCs (x10^3/uL, *n* = 40) [5.09–17.41]	14.6 (7.8, 21.0)	18.5 (13.1, 22.2) (*n* = 26)	7.9 (3.9, 14.0) (*n* = 14)	0.002*	0.022*
Increased WBCs	16 (40%)	14 (54%)	2 (14%)	0.019*	0.040*
Decreased WBCs	7 (17.5%)	2 (8%)	5 (36%)		
Band neutrophils (x10^3/uL, *n* = 40) [0–0.3]	0.6 (0, 1.8)	0.4 (0, 1.2) (*n* = 26)	1.2 (0.5, 7.4) (*n* = 14)	0.014*	0.040*
Increased bands	26 (65%)	15 (58%)	11 (79%)	0.187	0.206
Platelet count (x10^3/uL, *n* = 40) [52–518]	233.5 (168.3, 360)	251 (187.5, 67.8) (*n* = 26)	191.5 (140.8, 325.3) (*n* = 14)	0.178	0.206
Decreased plt	1 (2.5%)	1 (4%)	-	0.065	0.102
Increased plt	5 (12.5%)	4 (15%)	1 (7%)		
Body cavity fluid analysis (cytologically) (*n* = 41)					
Negative bacteria	12 (29%)	10 (40%)	2 (13%)	0.055	0.101
Positive bacteria	29 (71%)	15 (60%)	14 (88%)		
Fluid culture (aerobic) (*n* = 35)					
Positive	20 (57%)	11 (46%)	9 (82%)	-	-
Negative	15 (43%)	13 (54%)	2 (18%)		
Fluid culture (anaerobic) (*n* = 22)					
Positive	13 (59%)	9 (56%)	4 (57%)	-	-
Negative	9 (41%)	7 (44%)	2 (33%)		
CT findings					
Granuloma or abscess	5	4	1	-	-
Neoplasia	6	3	3		
Pneumothorax	9	6	3		
Pneumonia	13	10	3		
Pleural thickening	10	8	2		
Pleural effusion (*n* = 34)					
Unilateral	6 (18%)	3	3	-	-
Bilateral	28 (82%)	18	10		
Fluid volume					
Undefined	5 (12%)	2 (7%)	3 (19%)	0.075	0.103
Mild	10 (23%)	9 (33%)	1 (6%)		
Mild to moderate	7 (16%)	6 (22%)	1 (6%)		
Moderate	9 (21%)	6 (22%)	3 (19%)		
Moderate to large	4 (9%)	1 (4%)	3 (19%)		
Large	8 (19%)	3 (11%)	5 (31%)		
Treatment					
Non-surgical	22 (51.2%)	10 (37%)	12 (75%)	0.016*	0.042*
Surgical	21 (48.8%)	17 (63%)	4 (25%)		
Chest tube placement (non-surgical)					
Y	13 (59%)	6 (60%)	7 (58%)	-	-
*N*	9 (41%)	4 (40%)	5 (42%)		
Unilateral	8 (62%)	4 (67%)	4 (67%)	-	-
Bilateral	5 (38%)	2 (33%)	3 (43%)		

Adjusted *p*-values were calculated using the Benjamini-Hochberg Procedure with a false detect rate (FDR) threshold set at 0.05. The asterisk identifies that the *p*-values are significant at the 0.05 level.

Body cavity fluid analysis was performed in 41 dogs, with bacteria identified in 29 (71%) dogs through direct stained smears of the pleural fluid (Table 3). A mixed bacterial population of rods, cocci and/or filamentous-type organisms was observed in 25 (86%) of these cases, while in the remaining 4 cases only a single bacterial type was identified. Filamentous organisms were presented in 10 (34%) cases. Both intracellular and extracellular bacteria were identified in 27 (93%) cases. Among the 29 dogs with positive cytological findings, aerobic culture was submitted for 25 (86%) cases, with 16 (64%) dogs testing positive. Anaerobic culture was performed in 16 (55%) of dogs, and 12 (75%) of them tested positive. For the 12 cases without bacteria identified cytologically, 9 (75%) had aerobic culture results, with 5 (56%) yielding positive results; Anaerobic culture was performed for 6 dogs, with 1 (17%) testing positive. The most common aerobic bacterial isolates were *Escherichia coli* (*n* = 7) and *Streptococcus* spp. (*n* = 6); while *Peptostreptococcus anaerobius* and *Bacteroides* spp. were the most frequently isolated anaerobes. The specific bacterial isolates and their frequency are detailed in Table 4. A larger proportion of non-survivors (9/11, 81.82%) had positive aerobic fluid cultures compared to survivors (11/24, 45.83%); however, statistical significance was not evaluated due to the limited sample size. No other variables from the body cavity analysis showed significant differences between the survivor and non-survivor groups.

**Table 4:** Thoracic fluid aerobic and anaerobic culture results in dogs and cats with pyothorax.

Culture Method	Dogs		Cats	
	Bacterial organism	*n*	Bacterial organism	*n*
Aerobic	*Escherichia coli*	7	*Pasteurella multocida*	1
	Streptococcus spp.	6	*Pasteurella canis*	1
	Actinomyces spp.	4	Gram positive rod	1
	Pasteurella spp.	4	Gram negative rod	1
	Enterococcus spp.	3		
	*Bacteroides uniformis*	1		
	*Clostridium perfringens*	1		
	*Enterobacter cloacae*	1		
Anaerobic	*Peptostreptococcus anaerobius*	5	*Prevotella bivia*	1
	Bacteroides spp.	5	*Peptostreptococcus prevotii*	1
	Anaerobic gram-negative rod	2	*Bacteroides uniformis*	1
	*Clostridium perfringens*	1	*Bacteroides pyogenes*	1
	*Enterococcus faecium*	1	Anaerobic gram-positive rod	1
	*Escherichia coli*	1	Anaerobic gram-negative rod	1
	Fusobacterium species	1		
	*Mobiluncus mulieris*	1		
	*Porphyromonas gingivalis*	1		
	Prevotella species	1		
	*Streptococcus gallolyticus*	1		
	Actinomyces species	1		
	Anaerobic gram-positive cocci	1		

Thoracic radiographs were performed in 34 (79%) cases, with pleural effusion being the most common finding, reported in 29 (85%) cases. Other notable radiographic abnormalities included an alveolar pattern (*n* = 16, 47%) pneumothorax (*n* = 7, 18%), pneumonia/bronchopneumonia (*n* = 6, 18%), atelectasis (*n* = 5, 15%), and hepatomegaly (*n* = 4, 12%). Only 3 dogs underwent thoracic ultrasonography: one had focal thoracic ultrasonography for fine needle aspiration for a thoracic wall mass; another case revealed a pulmonary abscess; and one dog had a heterogeneous area of consolidation seen on the thoracic radiographs that was further evaluated by ultrasonography. CT was carried out in 17 (12%) dogs, with significant findings including pneumonia/bronchopneumonia (*n* = 13, 76%), pneumothorax (*n* = 9, 53%), and pleural thickening/pleuritis (*n* = 10, 59%). Abdominal radiographs and abdominal ultrasounds were performed in 12 dogs and 11 dogs, respectively. The characteristics of pleural effusion determined by imaging, were as follows: 28 (82%) dogs had bilateral pleural effusion, 6 (18%) had unilateral effusion with 4 (67%) on the left and 2 (33%) on the right. The affected side was not specified in 9 cases. The volume of pleural fluid was described as mild in 10 (23%) dogs, mild to moderate in 7 (16%), moderate in 9 (21%), moderate to large in 4 (9%), and large in 8 (19%). No significant difference was found between survivors and non-survivors regarding imaging findings. A summary of diagnostic imaging findings is presented in Table 5.

**Table 5:** A summary of imaging findings (radiographs, ultrasounds, and CT) in dogs with pyothorax.

Thoracic radiographs (*N* = 34)						CT (*N* = 17)			Abdominal imaging (*N* = 12)		
Thoracic structure	*n*	%	Extra-thoracic structure	*n*	%		*n*	%		*n*	%
Pleural effusion	29	85%	Hepatomegaly	4	12%	Granuloma or abscess	5	29%	Hepatomegaly	3	25%
Alveolar pattern	16	47%	Decreased cranial abdominal serosal detail	2	6%	Neoplasia	6	35%	Prostatomegaly	1	8%
Pneumothorax (or suspected)	7	21%	Subcutaneous emphysema	2	6%	Pneumothorax	9	53%	Gastric foreign material	2	17%
Pneumonia/bronchopneumonia	6	18%	Other findings (*n* = 1): air distended intestines, mineral opacity in the cranioventral abdomen, chronic kidney disease, pyloric foreign body, prominent mammary parenchyma			Pneumonia	13	76%	Splenomegaly	1	8%
Atelectasis	5	15%				Pleural thickening	10	59%	Spondylosis deformans	1	8%
Increased pulmonary opacity	3	9%							Osteoarthrosis	1	8%
Lung lobe retraction	2	6%							Developed mammary tissue	1	8%
lung consolidation	2	6%									
Pneumomediastinum (or suspected)	2	6%									
Small pulmonary blood vessels	2	6%									
Soft tissue opacity	2	6%									
Other findings (*n* = 1): lung contusion, unstructured interstitial pattern, dorsally elevated trachea, Bronchial pattern, pneumoperitoneum, pleuritis, esophageal foreign body, ill-defined heterogeneous area within lung field, chronic rib fracture											

#### 3.2.2 Cats

Hematology results (Table 2) were available for 5 cats. Of these, 2 (40%) cats were anemic (24.3 and 28.5%), and both were survivors (median 37.25, 24.3 to 49%). All surviving cats (*n* = 4) had a leukocytosis (median 26.97 ×10^3/uL, 17.57 to 88.87 ×10^3/uL) and an increase in band neutrophils (median 8.05 ×10^3/uL, 0.699 to 17.77 ×10^3/uL). Body cavity fluid analysis was performed in 7 cats. Bacteria was identified microscopically in 6 cases; with one case where bacteria were suspected but not confirmed. Five cases had aerobic culture submitted and 3 (60%) of them tested positive. Anaerobic cultures were performed in 4 cases with all being positive. *Pasteurella* spp. (*n* = 2) and *Bacteroides* spp. (*n* = 2) were the most common cultured aerobic and anaerobic bacteria, respectively (Table 4). Thoracic radiographs were performed in 4 cats, all following chest tube placement. No abdominal radiograph or ultrasonography were performed in this population. Seven cats had either CT (*n* = 4) or necropsy (*n* = 3) performed for further diagnosis. Bilateral pleural effusion was identified in 4 cats and unilateral effusion identified in 1. The volume of pleural fluid was described as mild (*n* = 1), moderate (*n* = 1), and large (*n* = 2); the characteristics of pleural effusion were undefined for 3 cats. Four cats had imaging findings consistent with pleuritis (*n* = 1), pleuropneumonia (*n* = 2), and a pulmonary abscess (*n* = 1).

### 3.3 Quick assessment tests (QATs)

#### 3.3.1 Dogs

QATs were performed on all dogs at the time of hospital admission (Table 6). The mean PCV was 47.5% ± 9.4%, and median (IQR) total solids measured 6.6 g/dL (6.0 to 7.7 g/dL). Dogs in the non-survivor group (median 6.3 g/dL, 6.0 to 6.8 g/dL) had a significantly (*p* = 0.023) lower measurement of total solids compared to those in the survivor group (median 7.2 g/dL, 6.4 to 8.0 g/dL). The median blood lactate and glucose concentrations on presentation, measured with a point-of-care device were 2.5 (1.7 to 3.5) mmol/L and 89 (74.0 to 115.0) mg/dl, respectively. Hyperlactatemia was present in 21 (50%) dogs, with 11 (11/16, 68.8%) classified as non-survivors, and 10 (10/26, 38.4%) as survivors. Initial blood lactate was significantly lower (*p* = 0.015) in survivors (median 2 mmol/L, 1.5 to 3.0 mmol/L) compared to non-survivors (median 3.35 mmol/L, 2.1 to 4.0 mmol/L). Four (4/43, 9.3%) dogs had dysglycemia with 1 case of hyperglycemia (non-survivor) and 3 cases of hypoglycemia (non-survivors [*n* = 2], survivor [*n* = 1]). Dysglycemia, analyzed as either a continuous variable (recorded values) or a categorical variable (hypoglycemia, normoglycemia, or hyperglycemia), did not differ significantly between surviving and non-surviving dogs. Recheck lactate concentrations the following morning were available for 35 dogs (26 survivors and 9 non-survivors), and the median measurement was 1.4 (0.9 to 1.8) mmol/L with 5 (13.9%) dogs being hyperlactatemic. The mean lactate delta for dogs with an initial elevated lactate concentration was −1.86 ± 1.02 mmol/L, and the mean lactate clearance was 51.9% ± 27.5%. There was no significant difference in lactate delta (*p* = 0.474) and lactate clearance (*p* = 0.226) between survivors and non-survivors. The median recheck glucose for 36 dogs was 106 (91 to 123) mg/dl with 1 case of hyperglycemia and 1 case of hypoglycemia. Recheck PCV, total solids, POC blood lactate, and glucose at 8 AM the following morning were not significantly different between groups (*p* > 0.05).

**Table 6:** Comparison of admission Quick Assessment Tests (QATs), recheck QATs, lactate delta, and lactate clearance in dogs with pyothorax between survivors and non-survivors.

QATs	Overall (*n* = 43)	Survivor (*n* = 27)	Non-survivor (*n* = 16)	*p*-value
PCV 1 (%) (*n* = 42)	47.5 ± 9.4	48.9 ± 9.3	45.1 ± 9.5 (*n* = 15)	0.208
TP 1 (g/dl) (*n* = 41)	6.6 (6.0, 7.7)	7.2 (6.4, 8.0) (*n* = 26)	6.3 (6.0, 6.8) (*n* = 15)	0.023*
BG 1 (mg/dl) (*n* = 43)	89 (72, 117)	89 (74.0, 115.0)	89.5 (70.0, 122.5)	0.97
Hyperglycemia 1	1 (2%)	-	1	0.192
Hypoglycemia 1	3 (7%)	1	2	
Lac 1 (mmol/L) (*n* = 42)	2.5 (1.7, 3.5)	2.0 (1.5, 3.0) (*n* = 26)	3.4 (2.1, 4.0)	0.015*
Hyperlactatemia 1	21 (50.0%)	10 (38.4%)	11 (68.8%)	0.044*
PCV 2 (%) (*n* = 36)	40.94 ± 8.92	41.22 ± 8.42	40.11 ± 10.78 (*n* = 9)	0.751
TP 2 (g/dl) (*n* = 35)	6.08 ± 1.35	6.19 ± 1.31	5.70 ± 1.50 (*n* = 8)	0.376
BG 2 (mg/dl) (*n* = 36)	106 (91, 123)	107.0 (96.0, 123.0)	91.0 (68.5,136.5) (*n* = 9)	0.221
Hyperglycemia 2	1	1	-	0.184
Hypoglycemia 2	1	-	1	
Lac 2 (mmol/L) (*n* = 35)	1.4 (0.9, 1.8)	1.2 (0.8, 1.8)	1.6 (1.4, 2.3) (*n* = 8)	0.064
Hyperlactatemia 2	5 (13.9%)	3 (11.1%)	2 (25%)	0.404
Lactate delta (mmol/L) (*n* = 16)	−1.86 ± 1.02	−2.01 ± 0.73 (*n* = 10)	−1.62 ± 1.43 (*n* = 6)	0.474
Lactate clearance (%) (*n* = 16)	51.90 ± 27.51	58.51 ± 21.01 (*n* = 10)	40.87 ± 35.24 (*n* = 6)	0.226

The asterisk identifies that the *p*-values are significant at the 0.05 level.

#### 3.3.2 Cats

In cats, the initial mean measurements of PCV and total solids were 46.57% ± 12.79% and 7 ± 0.5 g/dL. The mean blood lactate and glucose concentrations on admission were 4.64 ± 4.9 mmol/L and 137.75 ± 59.82 mg/dL, with hyperlactatemia observed in 5 (62.5%) cats including 2 survivors and 3 non-survivors, while dysglycemia was noted in 2 (25%) cats including 1 case of hyperglycemia (survivor) and 1 case of hypoglycemia (non-survivor). Recheck measurements the following morning were available for 5 cats, with a mean recheck blood lactate at 1.12 ± 0.16 mmol/L, and no cats showing abnormal lactate levels. The mean recheck blood glucose concentration was 134.8 ± 40.23 mg/dL, with 1 cat identified as hyperglycemic (survivor).

### 3.4 Diagnosis

#### 3.4.1 Dogs

An underlying cause was not identified in 23/43 (53%) of cases. In the remaining 20 cases, identified or suspected causes included migration of plant material (*n* = 3, 14%), penetrating foreign bodies (*n* = 3, 14%), abscesses (pulmonary abscess [*n* = 3] and pulmonary esophageal abscess [*n* = 1], 19%), neoplasia (*n* = 3, 14%), surgical site infection (*n* = 2, 10%), and 1 case each for trauma, esophageal perforation, lung lobe torsion, pneumonia (mycoplasma), and pyometra (2).

#### 3.4.2 Cats

The definitive underlying cause of pyothorax was not determined in any feline case. However, a pulmonary abscess was suspected in one case based on CT findings. Another cat had a history of going missing and had superficial wounds over the body when found. Septic inoculation was highly suspected in one case according to the results of necropsy.

### 3.5 Treatment and outcome

#### 3.5.1 Dogs

Among the cases, 22 dogs received medical therapy, while 21 underwent surgery. In the group with medical management, 13 dogs had a chest tube placed (unilateral [*n* = 8], bilateral [*n* = 5]). Surgical procedures performed included median sternotomy (*n* = 12, 57%), lung lobectomy (*n* = 8, 38%), exploratory thoracotomy (*n* = 5, 24%), esophagotomy (*n* = 2, 10%), and partial ventral mediastinectomy (*n* = 1, 5%). In-hospital antibiotics were administered in 39 cases. Ampicillin/sulbactam was the most common antimicrobial choice in this study (*n* = 35; 90%), followed by enrofloxacin (*n* = 18; 46%) dogs. Other antibiotics included clindamycin (*n* = 2), doxycycline (*n* = 1), ticarcillin/clavulanic acid (*n* = 2), trimethoprim/sulfamethoxazole (*n* = 1), ciprofloxacin (*n* = 2), amikacin (*n* = 2), and meropenem (*n* = 3). Multiagent therapy was prescribed in 23 dogs.

In total, 27 dogs survived, resulting in an overall survival rate of 62%, with a mean length of hospitalization (LOH) of 7.1 ± 2.6 days (range from 3 to 13 days). For dogs receiving medical management (*n* = 22), 10 dogs survived to discharge, 8 (36%) were euthanized, and 4 (17%) died. Survivors in the medical management group had a mean hospitalization duration of 5.8 ± 2.7 days (range from 3 to 12 days). In the surgical therapy group (*n* = 21), 17 dogs survived, yielding a survival rate of 81%. For survivors the mean duration of hospitalization was 7.8 ± 2.2 days (range from 5 to 13 days). For non-survivors, the median of the length of hospitalization prior to death was 2.3 ± 1.3 days (range from 1 to 6 days). The majority of non-surviving dogs (11/16, 68.8%) either died or were euthanized within the first 48 h following admission. A significant (*p* = 0.042) (Table 3) difference was found in survival rates between patients with surgical intervention (17/21, 81%) and those receiving medical management alone (10/22, 45.5%). Survivors who underwent surgery had a significantly (*p* = 0.016) (Table 7) longer length of hospitalization by 2.0 days compared to those managed medically. No other variables in terms of treatment showed a significant difference between survivors and non-survivors.

**Table 7:** Comparison of outcomes and length of hospitalization in dogs with pyothorax between survivors and non-survivors and between surgical and non-surgical management groups.

Variable	Survivor	Non-survivor	*p*-value
Survival [*n* (survival rate)]	27 (63%)	16 (37%)	-
Surgical	17 (81%)	-	-
Non-surgical	10 (45%)	-	-
LOH (days)	7.1 ± 2.6	-	-
Surgical	7.8 ± 2.2	-	0.016*
Non-surgical	5.8 ± 2.7	-	
Duration of Hospitalization (in-hospital death) (days)	-	2.3 ± 1.3	-

The asterisk identifies that the *p*-values are significant at the 0.05 level.

#### 3.5.2 Cats

All the cats received only medical management. Of the 8 cats, 6 had a chest tube placed, with 1 having bilateral placement. The antibiotics applied in the hospital included ampicillin/sulbactam (*n* = 5), enrofloxacin (*n* = 2), metronidazole (*n* = 2), and clindamycin (*n* = 1). Multi-agent therapy was prescribed in 5 cats. The survival rate was 50%, and the median duration of hospitalization for survivors was 6.5 days (range from 5 to 8 days).

### 3.6 Association between clinical factors and outcomes

Univariable logistic regression analysis was performed to investigate the associations between selected variables and in-hospital mortality. Significant variables identified between survivors and non-survivors included HCT, WBCs, band neutrophils, total solids on admission, POC blood lactate on admission, and treatment type (surgical vs. medical management). As shown in Table 8, WBCs were not associated with in-hospital mortality. The variables identified with statistically significant univariate analysis were then subjected to stepwise COX logistic regression to determine independent predictors of mortality. Total solids on admission and POC lactate concentration on admission were found to be predictive, with total solids negatively correlating and POC lactate positively correlating with in-hospital mortality. Dogs had 1.801 higher odds of mortality for every 1 mmol/L increase in lactate concentration (Table 8).

**Table 8:** Univariate and Stepwise cox regression analyses of in-hospital mortality in dogs with pyothorax.

Variables	Univariable regression		Stepwise Cox Regression	
	OR (95%-CI)	*p*-value	Coef	*p*-value
Lac 1	1.801 (1.052–3.084)	0.032*	0.117	0.006*
TP 1	0.416 (0.193–0.897)	0.025*	−0.202	0.002*
HCT	0.917 (0.850–0.990)	0.027*		
WBCs	0.962 (0.905–1.022)	0.211		
Bands	1.398 (1.034–1.892)	0.03*		
Treatment (surgical vs. non-surgical)	0.196 (0.050–0.775)	0.02*		

The asterisk identifies that the *p*-values are significant at the 0.05 level.

**Figure 1:**
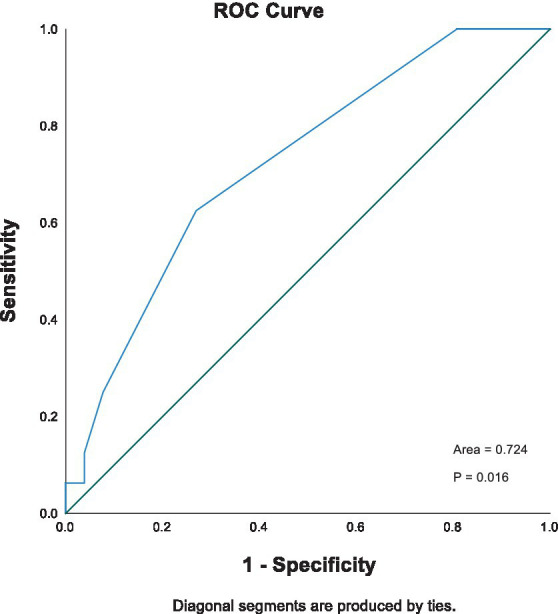
ROC of lactate (AUROC 0.724) for mortality prediction in dogs with pyothorax.

**Figure 2:**
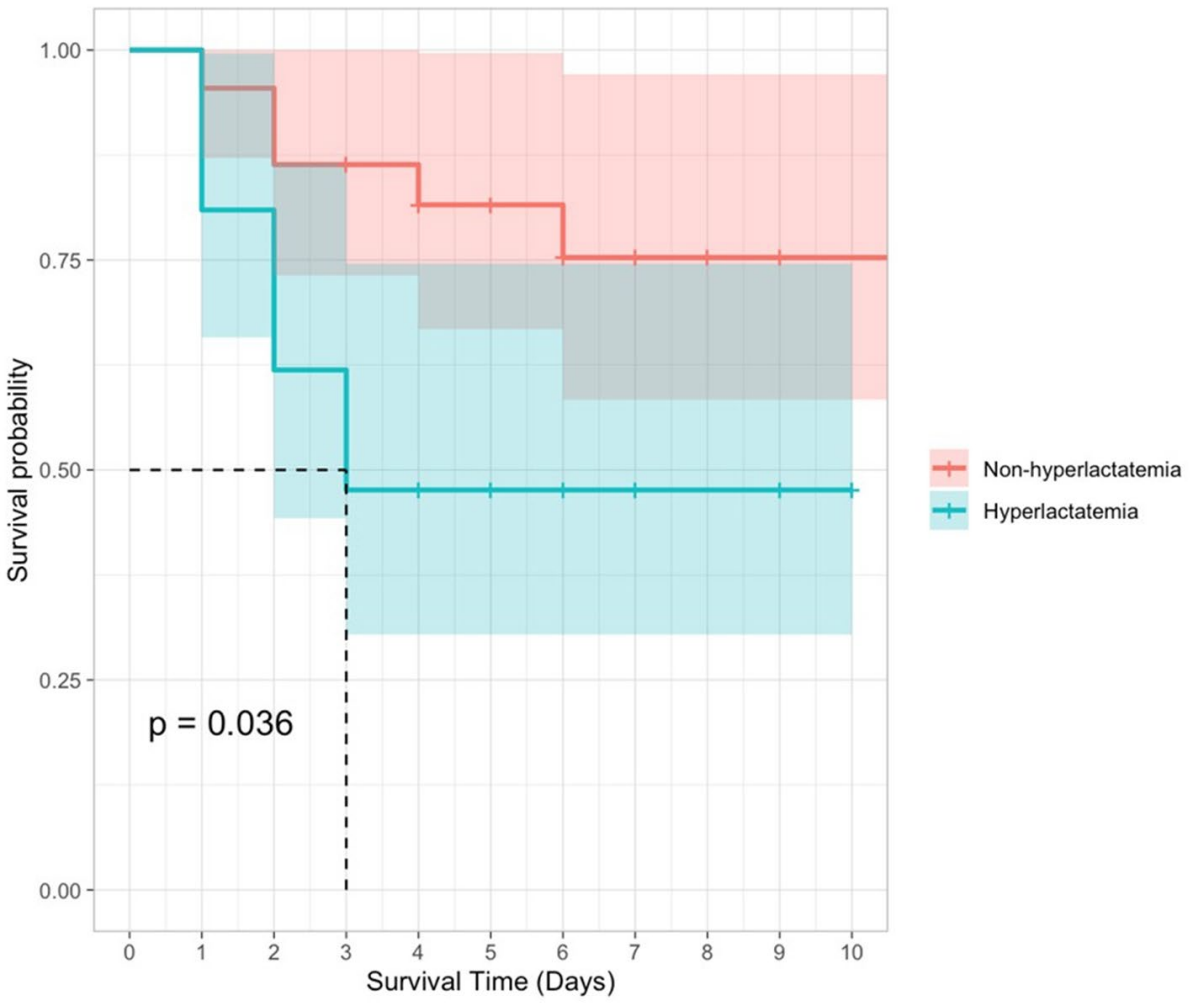
Kaplan–Meier survival curves for dogs with pyothorax by POC lactate concentration (hyperlactatemia vs. non-hyperlactatemia). 95% confidence intervals are represented by shaded areas.

**Figure 3:**
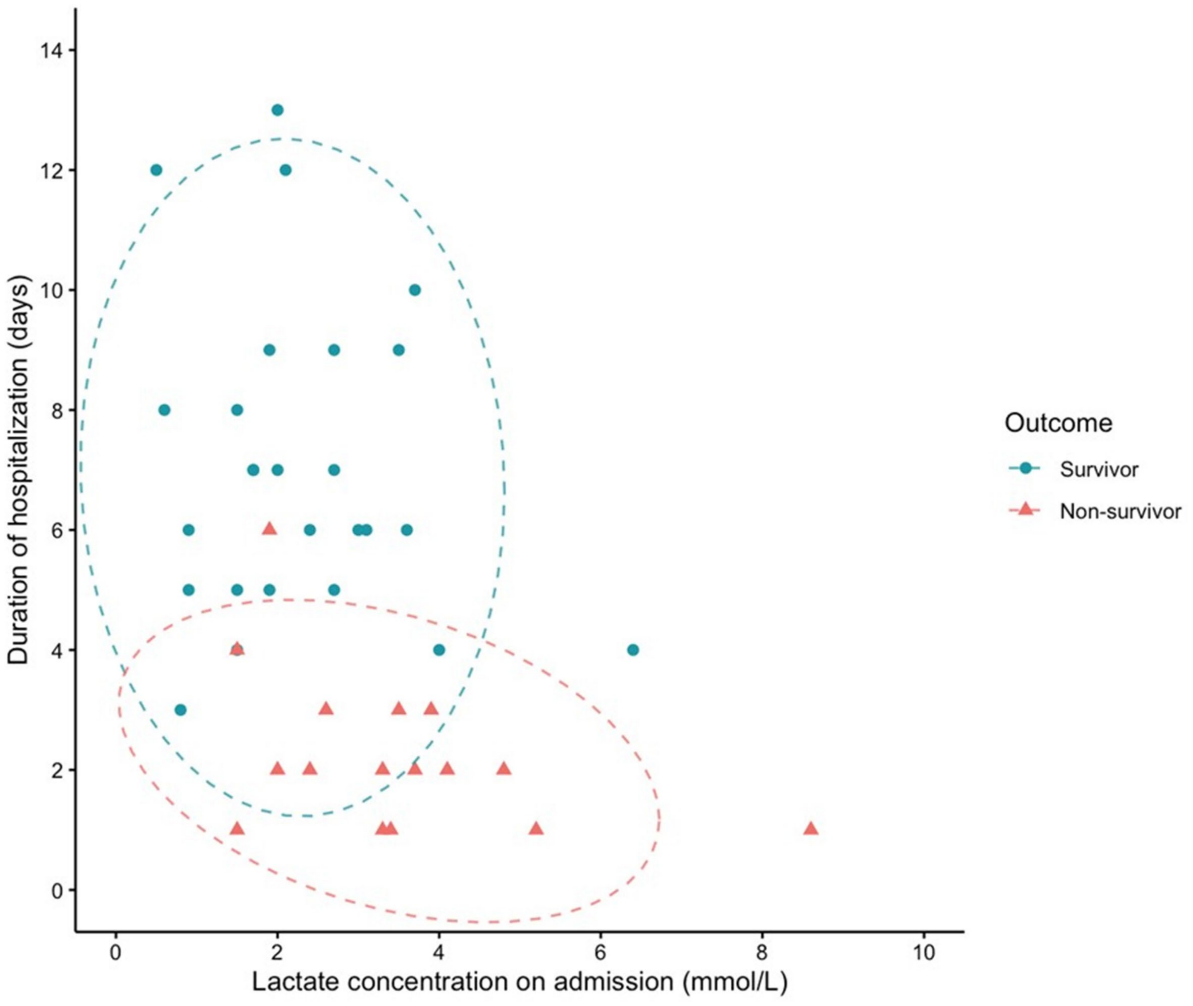
Scatterplot of POC lactate concentration on admission vs. duration of hospitalization (survival to discharge or time of death). 95% confidence ellipses are represented by dashed lines.

The receiver operating characteristic (ROC) curve was also applied to assess the predictive performance of initial blood lactate concentration. As seen in Figure 1, the AUROC (95% CI) for lactate concentration was 0.724 (0.568–0.879). The optimal cutoff for lactate to predict in-hospital mortality was 3.1 mmol/L, with a sensitivity of 0.625 and specificity of 0.808. Kaplan–Meier survival curves comparing survival in dogs with increased and normal POC lactate concentrations are provided in Figure 2. A scatterplot of POC lactate concentrations on admission compared to duration of hospitalization is provided in Figure 3.

## 4 Discussion

The aim of this retrospective study was to evaluate the prognostic value of point-of care blood lactate and glucose concentrations on admission, as well as their changes during the first 24 h of hospitalization, in dogs and cats diagnosed with pyothorax. Secondary objectives were to describe the signalment, history, physical examination findings, diagnostic findings, and in-hospital treatment, and to investigate their associations with survival.

Increased blood lactate levels have been found to have an association with severe disease states and increased hospital mortality in both humans and animals (11–16, 26). Blood lactate is a by-product of anaerobic glycolysis, and hyperlactatemia occurs when lactate formation exceeds its metabolism (27). Persistent hyperlactatemia has a significant association with in-hospital mortality in sick dogs in a variety of illness (15). In this study, hyperlactatemia was noted in 50% (21/43) of dogs and 62.5% (5/8) of cats. This is consistent with previous studies where the prevalence of hyperlactatemia for dogs and cats presented to the ER was 53–62% and 30–85% for dogs and cats, respectively (9, 14, 16). This finding are not surprising as hyperlactatemia is generally associated with onset, duration and severity of diseases (28).

As demonstrated in previous published studies evaluating other forms of severe illness (29, 30) admission lactate levels were also associated with in-hospital mortality in dogs with pyothorax. Statistical analysis in the current study found that dogs had 1.801 higher odds of mortality for every 1 mmol/L increase in lactate concentration. However, neither lactate delta nor lactate clearance showed an association with overall survival. In contrast, earlier studies found that both delta lactate and lactate clearance were different between survivors and non-survivors, with persistent hyperlactatemia associated with higher mortality in dogs with a variety of illness (15, 16). In our study, surviving dogs had a larger mean lactate delta (survivors −2.01 ± 0.73 mmol/L vs. non-survivors −1.62 ± 1.43 mmol/L) and higher mean lactate clearance (survivors 58.51 ± 21.01% vs. non-survivors 40.87 ± 35.24%) compared to non-survivors, though the differences were not statistically significant. Only 16 dogs had serial lactate measurements, patients that died or were euthanized before this recheck were not included in the analysis for lactate delta and clearance. This limited sample may have contributed to a type II error, masking potential association between changes in lactate (i.e., lactate clearance and lactate delta) and outcomes. In addition, blood lactate concentration was measured using hand-held POC devices, whereas earlier studies often utilized blood gas analyzers. While studies suggested that handheld hand-held lactate meters provide results in agreement with laboratory blood analyzers, variations in blood sampling techniques and sample handling may have influenced the results (31). Unless specified by the primary clinician, lactate (part of QATs) was rechecked at 8 AM the following morning during hospitalization, regardless of admission time, resulting in variability in the interval between measurements. The timing of lactate evaluations after admission also varied due to clinician judgment and resource availability. This lack of standardization, inherent to the retrospective nature of the study, could result in the lack of correlation between lactate delta and clearance and patient outcomes. Further studies with standardized timing and consistent intervals for serial lactate assessments are needed to better evaluate the utility of lactate delta and lactate clearance in dogs and cats with pyothorax.

The predictive value of lactate remains controversial in cats (14, 16, 17, 26). In this study, statistical analysis was not performed due to the small number of feline pyothorax cases. Further investigations are warranted to evaluate the association between blood lactate concentrations and outcomes in cats.

In this study, 2% (1/43) of dogs had hyperglycemia, and 7% (3/43) had hypoglycemia; in cats, 12.5% (1/8) had hyperglycemia and 12.5% (1/8) had hypoglycemia. The prevalence of dysglycemia was lower than reported in previous studies of critically ill dogs and cats, where 16–40% of dogs were hyperglycemic, 9% were hypoglycemic (32), and 54% of cats had hyperglycemia (33). These earlier studies focused on critically ill patients or those presented to ER services, differences in primary disease processes may account for the variability in abnormal blood glucose prevalence. Although patients receiving dextrose prior to blood sample collection were excluded in these studies, variability in blood sample handling, timing of blood glucose measurement, and measurement modalities may have influenced the glucose values.

Previously, studies have detected associations between dysglycemia and increased disease severity and mortality in dogs and cats with sepsis, dogs with congestive heart failure and traumatic brain injury, as well as critically ill cats (20, 22, 33, 34). In contrast, our study failed to demonstrate a significant relationship between glucose concentration and survival outcome. This could relate to the small number of patients with abnormal glucose levels, which limits the statical power of the analysis, as well as the specific disease focus of our population. Although emerging veterinary studies suggest that the development of dysglycemia may be associated with negative outcomes, it is unknown whether abnormal glucose concentration in animals with systemic disease has a detrimental consequence or if it is a marker for disease severity (32). In human medicine, glycemic regulation has been associated with improved outcomes, and treatment is often warranted due to the detrimental effect of abnormal glucose concentrations (35). Future research is needed to better understand the clinical relevance of hyperglycemia and hypoglycemia and to assess the role of glycemic control in veterinary patients.

The overall survival rate in this study was 63 and 50% for dogs and cats, respectively. The prognosis for canine and feline pyothorax is variable. Reported overall survival rates were 83% in dogs (range 29–100%) and 62% in cats (range 8–100%) (36). In the current study, dogs managed surgically had a significantly higher survival rate compared to those receiving medical management. However, dogs who underwent surgical procedures had a significantly longer length of hospitalization than those managed medically. This is likely due to cases that converted to surgical treatment if no improvement following medical management was observed, or severe cases that were stabilized before being considered suitable candidates for anesthesia and surgery (37, 38). Previous studies regarding the correlation between surgical versus medical management and outcomes have conflicting results. Two previously reported studies showed no difference between surgical and medical management regarding long-term outcomes (6, 9). Others indicated better short- and long-term outcomes with surgery (4, 6, 10). Due to the retrospective nature of this study, factors that might have affected the treatment plans were not evaluated, such as perceived illness severity, stability for anesthesia and surgery, and owner’s election for euthanasia because of financial constraints, perceived poor prognosis, and risk of recurrence. Additionally, long-term outcome could not be evaluated. There is no consensus or guidelines regarding treatment in canine and feline pyothorax, studies with a larger population are indicated to investigate the association between surgical versus medical management and outcomes.

In this study, the measurement of TS on admission was significantly associated with the survival outcome at both the univariable and multivariable levels. TS is used as an estimate for total protein concentration in veterinary medicine. Any cause of protein loss can lead to a decreased value of TS. We speculated that the lower TS in dogs that died is likely a result of an acute phase reaction with downregulation of albumin synthesis in coordination with upregulation of inflammatory cytokines, increased vascular permeability, and loss of protein into the pleural effusion (39–42). In previous studies, total plasma protein was identified as a prognostic marker in dogs with hemangiosarcoma, hemoabdomen, and immune mediated hemolytic anemia (IMHA) (41, 43, 44). Further studies are needed to confirm the clinical significance and the prognostic utility of TS in canine pyothorax, given the mechanism leading to a decreased total protein among diseases may be variable.

Dogs that died or were euthanized had significantly lower WBC counts and higher band neutrophils. The presence of leukopenia with neutrophilic left shift in dogs that died was probably secondary to the severe sepsis with more advanced disease process, which could complicate treatment and worsen the outcome. This correlated to results of previous studies, a left-shift leukogram has been associated with a poorer prognosis in dogs including longer hospitalization, increased treatment costs, and higher risk of mortality in several conditions (40, 45).

While a significant difference was found in hematocrit between dogs in the survivor and non-survivor groups in this study, no significant difference was noted in on-admission packed cell volume (PCV). One dog in the non-survivor group had a notable low HCT of 16.3% but the PCV of this patient was not measured. When this patient was excluded from analysis, neither HCT nor PCV showed significant difference between the survivor and non-survivor groups. This dog had a history of pyometra, and the hematological results showed leukocytosis with a left shit, ultimately experiencing cardiopulmonary arrest during thoracostomy tube placement. The observed anemia in this case is likely clinically accurate and is speculated to be associated with the severity of the underlying disease process.

Non-surviving cats in this study had lower body temperatures, lower heart rates, and higher respiratory rates than survivors. Cats with sepsis or septic shock typically display bradycardia, hypotension and hypothermia, rather than experience the hyperdynamic state seen in dogs and people, although this is not fully understood (46). The presence of hypothermia and bradycardia likely indicates severe illness. Tachypnea in non-survivors may result from more pleural effusion or pulmonary parenchymal disease. However, the small number of cats precluded statistical validation of these differences, further studies with larger sample sizes are needed.

This is a single-institution study, and the findings may not be generalizable to other settings. Several other limitations arise from its retrospective nature. One key limitation was the small sample size, particularly in the cat population, where only one non-surviving cat had hematological tests performed. This severely limited the ability to investigate potential meaningful differences between survivors and non-survivors. The timing of blood tests performed was also variable and inconsistent across the population. Diagnostic approaches, medical management, procedures and medical records were not standardized. Specific make and models of point-of-care analyzers could not be standardized over the 10-year period of this study. In addition, not all animals had diagnostic tests performed, due to financial constraints or perceived potential outcome, potentially introducing selection bias. Cases in which dogs and cats lacked available POC lactate and glucose concentrations due to the judgment of clinicians or the availability of resources were excluded from the study. These cases may have represented either sicker patients with poor prognoses or more stable patients with higher survival likelihood, the current study is limited by their exclusion. Furthermore, dogs and cats that died and those that were euthanized were grouped together, the negative survival outcome was consequently confounded by owners’ financial and personal decisions. It is not possible to determine whether the euthanized animals might have survived with continued care. In most cases, the decision to euthanize was prompted by severe clinical signs or worsening disease. However, some euthanized patients may have survived with continued care, and their inclusion in the non-survivor group may reduce the ability to identify potential differences between survivors and non-survivors. Lastly, some dogs classified as survivors may have terminal conditions, such neoplasia (3/27, 11% of dogs), as the study lacked follow-up data after discharge. These dogs may have been misclassified as survivors, further limiting the accuracy of survival outcomes.

In conclusion, point-of-care blood lactate concentration measured at admission may have a prognostic value in predicting outcomes for dogs with pyothorax. Due to the limitations of this retrospective study, additional large-scale prospective studies with standardized sampling intervals, particularly for lactate clearance, in both dogs and cats are needed to corroborate these findings before any changes in clinical practice can be recommended.

## Data Availability

The original contributions presented in the study are included in the article/supplementary material, further inquiries can be directed to the corresponding author.
